# Status of HBsAg seroprevalence in 15 million rural couples in China: a cross-sectional study

**DOI:** 10.1038/srep42822

**Published:** 2017-02-21

**Authors:** Long Zhang, Yuan-Yuan Wang, Yan-Jie Huang, Qiao-Mei Wang, Kenrad E. Nelson, An-Qi Wang, Hai-Ping Shen, Xiao-Li Liu, Yi-Ping Zhang, Dong-Hai Yan, Zuo-Qi Peng, Hong-Guang Zhang, Ya Zhang, Jun Zhao, Yan Wang, Ying Yang, Yuan He, Ji-Hong Xu, Du-Jia Liu, Tong-Jun Guo, Xiao-Na Xin, Hong Zhou, Xu Ma

**Affiliations:** 1 Environmental and Spatial Epidemiology Research Center, National Human Genetic Resources Center, Beijing, China; 2 Department of Epidemiology, Johns Hopkins Bloomberg School of Public Health, Baltimore, Maryland, United States; 3 National Research Institute for Family Planning, Beijing, China; 4 Graduate School of Peking Union Medical College, Beijing, China; 5 Department of Health Policy and Management, Johns Hopkins Bloomberg School of Public Health, Baltimore, Maryland, United States; 6 Department of Maternal and Child Health, National Health and Family Planning Commission of the PRC, Beijing, China; 7 Department of Child, Adolescent and Women’s Health, School of Public Health, Peking University, Beijing, China; 8 Key Laboratory of Reproductive Health, Ministry of Health, Beijing, China

## Abstract

A cross-sectional analysis of prevalence of hepatitis B virus infection (HBV) among rural couples was conducted between 2010 and 2014. Serologic HBV markers, including hepatitis B surface antigen (HBsAg) and e antigen (HBeAg), were tested. Primary outcome of interest comprised HBsAg positivity in couples (both positive: F+M+, only wife positive: F+M−, only husband positive: F−M+), and secondary outcome consisted of prevalence and risk factors of HBsAg positivity among husbands or wives. Of 14,816,300 couples included, 0.7% were F+M+; 6.3% were F−M+; 4.4% were F+M−, resulting in the overall seroprevalence of 11.4%. Individually, 6.1% were HBsAg positive with a higher rate seen in husbands (7.0%) than in wives (5.2%). Wife’s HBeAg(+)/HBsAg (+) (AOR = 2.61), HBeAg(−)/HBsAg (+) (AOR = 2.23), positivity of syphilis (AOR = 1.50), living in a high-risk region (AOR = 1.46) were significantly predictors of HBsAg positivity in husbands. Prevalence and predictors of HBsAg positivity in wives had similar results. Our data show a high burden and discordant pattern of HBV infection in rural couples, and partner’s double positivity of HBeAg and HBsAg was the most significant factor of HBV infection in couples. A comprehensive strategy that emphasizes vaccination and education is needed.

Hepatitis B virus (HBV) infection and its related complications remain a primary public health threat globally^1^. More than 240 million people were chronically HBV infected with 600,000 deaths per year related to HBV^1^,^2^. Due to enormous population base (1,357 million in 2013)^3^ and continual burden of HBV infection (7.2% in 2006)^4^, China has been known as the primary drive of global prevalence of HBV infection, accounting for approximate one third of global burden^1^. Mother to children transmission (MTCT) is the primary mode of transmission of HBV in China^5^, and Chinese government has primarily focused on reduction of new infections among infants and young age groups over the past decades by expanding HBV immunization and screening pregnant women for HBV^6^. With considerable success of this immunization campaign^7^, the prevalence among children aged <5 years has been reduced to 1.0% in 2006, a 90% reduction compared to the level in 1992^4^. Although infections have been much controlled among young age groups, the number of reported HBV infections in adults aged >20 years increased by 22%, from 740 thousands in 2004 to 903 thousands in 2014^8^.

For adults, sexual exposure and percutaneous contact to infected blood are the two major sources of new HBV infection^9^. HBV can be spread efficiently through sexual contact among heterosexual partners^10^. American CDC reported that 39% of new HBV infections resulted from heterosexual transmission^9^. Data on estimates of infections among heterosexual adults in developing countries is scarce given the prevailing view of its trivial role. In China, adults at risk for HBV infection, such as susceptible heterosexual partners of people with HBV infection, have not been prioritized for intervention despite uprising reported HBV infections in adults. It is of importance to provide evidence of prevalence of HBV infection and evaluate its risks of transmission among adult population in order to inform HBV prevention policy in China, and to provide insight into global HBV control in adults.

In this study, we aimed to report seroprevalence of HBsAg and to evaluate risks of prevalent HBV infection among rural couples, using data from the National Free-pregnancy Checkups (NFPC) program in China.

## Methods

### Study design and study population

A cross-sectional study was conducted among rural couples aged 20–49 years who planned to conceive within next six months between January 2010 and December 2014, using data from the National Free Pre-conception Check-up (NFPC) program. The NFPC program, sponsored by the Chinese National Health and Family Planning Commission and Ministry of Finance, is a national free medical examination service in conjunction with counselling services and directing to treatment, aiming to improve quality of conception and reduce risks of birth defects. The program began in 2010 and gradually scaled up to the entire country in 2014. Rural couples were defined as rural residents based on their household registration, and they voluntarily participated in this program if they planned to conceive after marriage, comprising a convenience sample in present study.

Before enrollment, program procedures and study purposes had been explained to each participant and a written informed consent had been obtained. The program procedure includes 1) an interviewed-administered questionnaire that collects data on demographics, medical history and assessment of environmental and behavioral exposure; 2) physical examination; 3) blood draw for laboratory testing including serologic markers of hepatitis B such as hepatitis B surface antigen (HBsAg), e antigen (HBeAg), core antibody (HBcAb, current or past infection) and surface antibody (HBsAb), sexual transmitted infection (STI) including syphilis, marker for liver damage including alanine aminotransferase (ALT) test and other testing related to prenatal care. Local laboratories tested serum samples for these biomarkers with reagent kits selected on their preferences (ELISA for HBV serologic markers; mostly ELISA for syphilis). National Center of Clinical Laboratory was responsible to inspect local reagent kits using a standard reagent for quality control (Abbott Part, IL, USA). Participants with positive testing results were consulted for preventive and clinical intervention. The Institutional Research Review Board at the National Research Institute for Family Planning had approved study protocols and forms, in accordance with the relevant guidelines and regulations.

### Definition of outcomes

The primary outcome was the status of HBsAg positivity in couples (commonly indicative of chronic HBV infection)^2^. Based on individual status of HBsAg positivity, couple’s status was categorized to: both wife and husband were HBsAg positive (wife (F) positive (+) husband (M) positive (+): F+M+); only wife was HBsAg (F+M−); only husband was HBsAg positive (F−M+); and neither were positive (F−M−). Secondary outcome of interest comprised positivity of HBsAg among either husbands or wives.

### Definition of covariates

Covariates included age (20–24, 25–29, and 30–49 years), education (≤primary, junior, senior, and ≥college), positivity of syphilis antibody testing (negative, positive), positivity of HBeAg (negative, positive), elevation of ATL (wife’s ALT > 38 U/I or husband’s ALT > 60 U/I; no, yes), region (based on prevalence of HIV among female sex workers in the region^11^ where participants lived, categorized as HIV low-risk (prevalence of HIV <1%), medium-risk (1–5%) and high-risk region (>5%)).

### Statistical analysis

Characteristics of study population were categorized using proportions in the entire cohort and by couple’s status of HBsAg positivity. Treating couples as analytical units, we used adjusted multinomial logistic regression models to explore risks of couple’s status of HBeAg positivity, including F+M+, F−M+ and F+M−. Adjusted variables included age, education, positivity of syphilis and region. Individual risks of HBsAg positivity (either in wives or in husbands) were explored using multivariate logistic regression models with considering their partner’s risk factors, including age, education, positivity of syphilis antibody, status of HBeAg and HBsAg, and region. Adjusted odds ratio (AOR) were used to present risks of each outcome. A 95% confidence interval (CI) that does not contain the null value was considered to reach statistical significance. All of statistical analyses were completed in Stata 13.1 (College Station, Texas, USA).

## Results

### Description of study population

**Figure 1:**
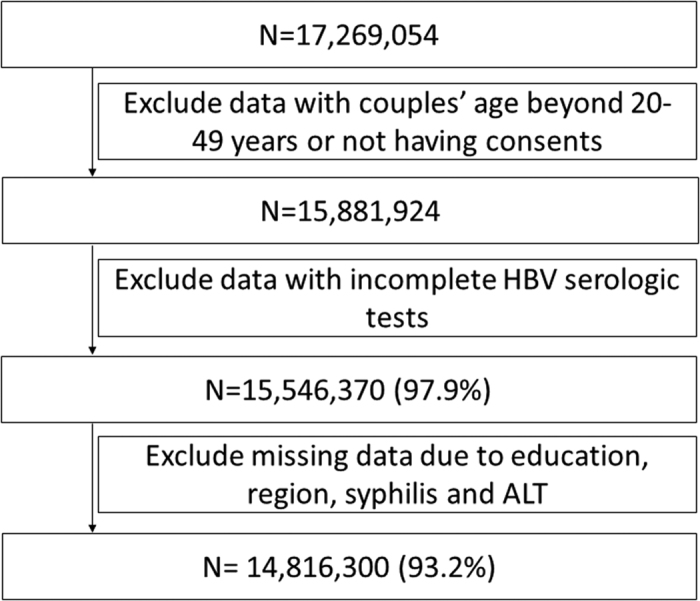
Flowchart of Study Population.

A total of 17,269,054 couples participated in NFPC program between January 2010 and December 2014. After exclusion of those whose age were beyond 20–49 years or who had not provided a written consent, 15,881,924 were left. Of them, 15,881,924 (97.9%) had completed all of HBV serologic tests and 14,816,300 (93.2%) had complete data regarding demographics and other tests (Fig. 1).

Majority of wives were aged 20–24 years (41.2%) and had junior schooling (64.4%). 0.4% were syphilis positive. 1.5% had positivity of HBeAg (28.8% among HBsAg positive wives) and 6.5% had ALT > 38 U/I (15.2% among HBsAg positive wives). 44.8% lived in a HIV-low risk region. Characteristics of husbands accord with their wife’s features. Distribution of couple’s characteristics varied by status of HBsAg positivity. For example, F+M+ couples were most likely to be syphilis positive compared to those who were F−M− (1.61% vs 0.57%). Age and education appeared to be less correlated with status of couple’s HBsAg positivity (Table 1).

### Seroprevalence of HBsAg

Of 14,816,300 couples, 109,562 (0.74%) were F+M+; 925,489 (6.3%) were F−M+; 655,015 (4.4%) were F+M−, resulting in the overall seroprevalence of 11.4% (Table 1). Among these affected couples, 93.5% had discordant status of HBsAg positivity.

**Figure 2:**
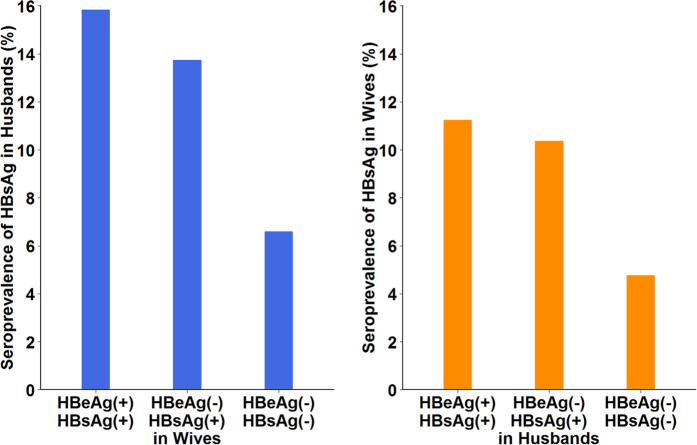
Prevalence of HBsAg Positivity among Individuals by Partner’s HBV Serostatus.

Individually, the overall seroprevalence of HBsAg is 6.1% with a higher rate in husbands than in wives (7.0% vs 5.2%). Stratified by partner’s HBV serostatus, we found 15.8%, 13.7%, and 6.6% of husbands were infected with HBV if their wives were HBeAg(+)/HBsAg(+), HBeAg(−)/HBsAg(+) and HBeAg(−)/HBsAg(−), respectively. Similarly, 11.2%, 10.4%, and 4.8% of wives were infected with HBV if their husbands were HBeAg(+)/HBsAg(+), HBeAg(−)/HBsAg(+) and HBeAg(−)/HBsAg(−), respectively (Fig. 2.). A similar trend was found when examining HBcAb and HBsAb. 24.5%, 19.9%, and 10.3% of husbands were HBcAb positive, and 48.4%, 39.8%, and 29.8% were HBsAb positive if their wives were HBeAg(+)/HBsAg(+), HBeAg(−)/HBsAg(+) and HBeAg(−)/HBsAg(−), respectively. Prevalences of HBcAb and HBsAb in wives resembled results found in husbands.

### Risk Factors for HBsAg Positivity in Couples

In the multivariate model, couple’s positivity of syphilis (AOR = 2.69, 95% CI 2.57–2.82) and living in a HIV high-risk region (AOR = 1.53, 95% CI 1.51–1.56) were significantly associated with higher odds of F+M+. Similarly, couple’s syphilis positivity (AOR = 1.73, 95% CI 1.69–1.76) and living in a HIV high-risk region (AOR = 1.50, 95% CI 1.49–1.51) were associated with an increased risk of F−M+. Risk factors for F+M− followed a similar pattern. Although wife’s age and education were significantly associated with each outcome, their point estimates were not very different from the reference group (Table 2).

### Risk Factors for HBsAg Positivity among Individuals

**Figure 3:**
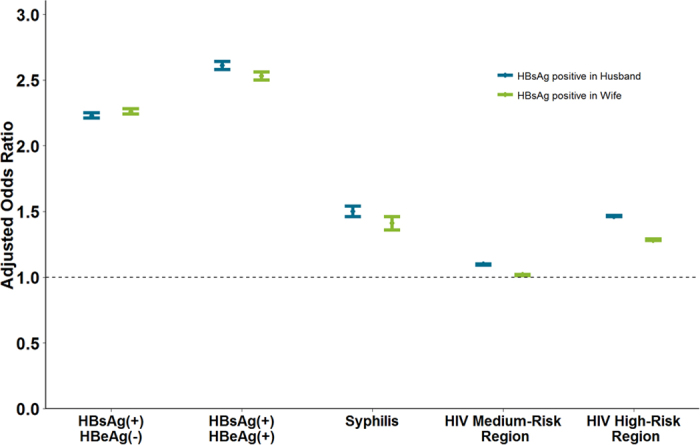
Multivariate Logistic Regression for HBsAg Positivity among Individuals. Individual (husband or wife)’s status of HBsAg positivity was modelled and their partner’s characteristics were analyzed as covariates. Blue bars refer to husband’s HBsAg positivity, and green bars refer to wife’s HBsAg positivity. Partner’s characteristics are displayed along with x-axis.

The multivariate model shows that wife’s HBeAg (+)/HBsAg(+) (AOR = 2.61, 95% CI 2.58–2.64), HBeAg (−)/HBsAg(+) (AOR = 2.23, 95% CI 2.21–2.25), positivity of syphilis (AOR = 1.50, 95% CI 1.46–1.54), living in a high-risk (AOR = 1.46, 95% CI 1.46–1.47) and medium-risk (AOR = 1.10, 95% CI 1.09–1.10) region were significantly associated with increased odds of being HBsAg positive in husbands. Unexpectedly, low wife’s education was protective. Similarly, HBsAg positivity in wives was more likely to occur among those whose husbands were HBeAg (+)/HBsAg(+) (AOR = 2.53, 95% CI 2.50–2.56), HBeAg (−)/HBsAg(+) (AOR = 2.26, 95% CI 2.24–2.28), syphilis positive (AOR = 1.41, 95% CI 1.36–1.46) and living in a HIV high-risk region (AOR = 1.28, 95% CI 1.28–1.29) (Fig. 3).

## Discussion

Our study demonstrates that 11.4% of rural couples who planned to conceive in China were affected by HBV infection and most of these infections were discordant, which may expose seronegative partner to a higher risk of infection. We found that people living with a HBV infected partner were more likely to have current HBV infection, to experience past or ongoing HBV infection, and to develop immune protection, suggesting closeness of married couples can risky for HBV transmission without appropriate preventive measures. Positivity of syphilis and living in a HIV high-risk region may also play a role in increased risk of HBV infection in couples.

The burden of HBV infection in this rural population, 6.1%, was slightly lower than the level found in 2007 national survey in China, 7.2%^4^. Treating couples as the analytical unit, we found 11.4% of them had at least a member with HBV infection. Most of these affected couples (93.5%) were one HBsAg positive and another HBsAg negative, providing a possibility of HBV transmission between couples. The discordant pattern of infections in couples also suggests that most of them were likely to be infected at birth or in their early childhood and carried their HBV infections into adulthood given the fact that up to 40–50% of new infections are acquired through MTCT in China^12^.

We found people’s active HBV infection was significantly associated with more than 2-fold risk of acquiring HBV infection among their partners. Similarly, people living with a partner who was HBeAg and HBsAg positive had higher likelihood of past or ongoing infection, and a higher rate of immune protection. All suggested an increased risk of transmission among discordant couples, likely through sexual route. Of note, most of HBV infection in adults is acute and self-limited, and 95% of infection can be resolved^9^, which is why we observed a much higher rate of past or ongoing infection and immunity. However, the ability of clearing a virus in adults would not guarantee a complete protection from HBV infection, and repeated exposure to a virus before developing immunity can be risky for transmission in couples.

The risk of HBV transmission in couples may further increase in China given our observation that 26% of HBsAg positive husbands and 29% of HBsAg positive wives were HBeAg positive, and 15.5% of husbands and 15.1% of wives carrying HBsAg had ALT > 60 U/I and ALT > 38 U/I, respectively, an indication of being in the immune-active phase although a clinical confirmation is needed^13^. Furthermore, among HBsAg positive wives, few of them (10%) were aware of their HBV status and even fewer (0.22%) had antiviral interventions despite more people (7.4%) in need of treatment (data not shown). Insufficient awareness and lack of antiviral treatment may pose an additional risk of HBV infection in couples.

Acquisition of HBV infection in couples has also been linked to partner’s positivity of syphilis and living in the HIV high-risk region. This resonated with findings from a recent study, which reported that uprising incidence of HBV infection in adults was correlated with that of other sexually transmitted diseases in China (including HIV, syphilis and gonorrhea)^14^. This evidence suggests a role of syphilis in facilitating the sexual transmission of HBV infection, reminiscent of a synergic role of syphilis in acquisition of HIV^15^. The definite relation between these two diseases warrants a further investigation.

Despite its high efficiency of spread^10^, HBV infection by sexual exposure or close contact has not been commonly recognized yet in China, and preventive measures to protect seronegative partners of people with HBV infection is far from optimum. A Hong Kong study conducted among 1623 pregnant Chinese women showed that although most of them realized the perinatal transmission and prevention of HBV infection by vaccination, 47.1% lacked of knowledge of sexual transmission of HBV infection^16^. According to the 2006–2010 National Guidelines for Hepatitis B Prevention and Treatment^6^, the main focuses were placed on increasing coverage of hepatitis B immunization in infants, especially timely birth dose. However, preventive measures among high risk groups, such as partners and household contacts of persons with HBV infection^9^, were not highlighted.

Of note, couples in our study are the ones who planned to conceive in near future, which may complex the HBV prevention due to unprotected sex. Although the NFPC program provides rural couples with valuable opportunities to screen HBV infection and to orient them to further clinical interventions, resources are still limited, such as vaccination of HBsAg negative partner of people with HBV infection, and programmes to increase access to information regarding benefits of vaccination and risks of HBV infection. To eliminate transmission of HBV infection, it is critical to implement comprehensive immunization strategies including adults at risk for HBV infection in addition to young age groups.

The strengths of the present study include an utmost large sample of rural populations in China and capturing people at risk for HBV infection. To our knowledge, this is the first study which explored HBV infection in couples, which provided a unique ability to evaluate the pattern of HBV infection between couples. Limitations are inability to be generalized to the entire population, lack of measurement of individual behavioral risks that may contribute to transmission, and lack of assessment of HBV DNA levels.

In conclusion, our data show a high burden of HBV infection in rural couples who planned to conceive. Most of HBV infections are discordant, which means a vast proportion of couples may be at risk for HBV infection. We predict that with the successful implementation of HBV immunization among infants and young age groups, new HBV infections in China will mostly come from transmission in adults, especially in discordant couples. A strong and comprehensive strategy that emphasizes vaccination of adults at risk for HBV infections and education on risk factors of HBV transmission should be scaled up immediately.

## Additional Information

**How to cite this article:** Zhang, L. *et al*. Status of HBsAg seroprevalence in 15 million rural couples in China: a cross-sectional study. *Sci. Rep.* **7**, 42822; doi: 10.1038/srep42822 (2017).

**Publisher's note:** Springer Nature remains neutral with regard to jurisdictional claims in published maps and institutional affiliations.

## Figures and Tables

**Table 1:** Characteristics of Study Population.

	Entire cohort N (%)	F−M− N (%)	F+M+N (%)	F−M+N (%)	F+M− N (%)
Total	14,816,300	13,126,234 (88.59)	109,562 (0.74)	925,489 (6.25)	655,015 (4.42)
Wife’s age					
20–24 years	6,098,107 (41.16)	5,402,877 (41.16)	45,410 (41.45)	385,502 (41.65)	264,318 (40.35)
25–29 years	5,667,707 (38.25)	5,017,326 (38.22)	42,694 (38.97)	356,193 (38.49)	251,494 (38.40)
30–49 years	3,050,486 (20.59)	2,706,031 (20.62)	21,458 (19.59)	183,794 (19.86)	139,203 (21.25)
Wife’s education					
≥college schooling	1,920,109 (12.96)	1,684,211 (12.83)	14,177 (12.94)	135,357 (14.63)	86,364 (13.19)
senior schooling	2,523,227 (17.03)	2,221,575 (16.92)	19,641 (17.93)	167,656 (18.12)	114,355 (17.46)
junior schooling	9,547,592 (64.44)	8,493,890 (64.71)	68,957 (62.94)	569,443 (61.53)	415,302 (63.40)
≤primary schooling	825,372 (5.57)	726,558 (5.54)	6,787 (6.19)	53,033 (5.73)	38,994 (5.95)
Couple’s syphilis					
negative	14,723,621 (99.37)	13,050,851 (99.43)	107,795 (98.39)	915,990 (98.97)	648,985 (99.08)
positive	92,679 (0.63)	75,383 (0.57)	1,767 (1.61)	9,499 (1.03)	6,030 (0.92)
Wife’s syphilis					
negative	14,762,220 (99.63)	13,081,969 (99.66)	108,280 (98.83)	920,917 (99.51)	651,054 (99.4)
positive	54,080 (0.37)	44,265 (0.34)	1,282 (1.17)	4,572 (0.49)	3,961 (0.6)
Husband’s syphilis					
negative	14,769,846 (99.69)	13,089,199 (99.72)	108,300 (98.85)	919,849 (99.39)	652,498 (99.62)
positive	46,454 (0.31)	37,035 (0.28)	1,262 (1.15)	5,640 (0.61)	2,517 (0.38)
Couple’s HBeAg					
negative	14,335,564 (96.76)	13,126,234 (100)	57,267 (52.27)	682,250 (73.72)	469,813 (71.73)
positive	480,736 (3.24)	NA	52,295 (47.73)	243,239 (26.28)	185,202 (28.27)
Wife’s positivity of HBeAg					
negative	14,596,265 (98.51)	13126,234 (100)	74,729 (68.21)	925,489 (100)	469,813 (71.73)
positive	220,035 (1.49)	NA	34,833 (31.79)	NA	185,202 (28.27)
Husband’s positivity of HBeAg					
negative	14,542,284 (98.15)	13,126,234 (100)	78,785 (71.91)	682,250 (73.72)	655,015 (100)
positive	274,016 (1.85)	NA	30,777 (28.09)	243,239 (26.28)	NA
Couple’s elevated ALT					
no	12981616 (87.62)	11648063 (88.74)	78633 (71.77)	735111 (79.43)	519809 (79.36)
yes	1834684 (12.38)	1478171 (11.26)	30929 (28.23)	190378 (20.57)	135206 (20.64)
Wife’s ALT > 38 U/I					
no	13,856,007 (93.52)	12,342,305 (94.03)	91,833 (83.82)	865,488 (93.52)	556,381 (84.94)
yes	960,293 (6.48)	783,929 (5.97)	17,729 (16.18)	60,001 (6.48)	98,634 (15.06)
Husband’s ALT > 60 U/I					
no	13,826,148 (93.32)	12,341,764 (94.02)	92,638 (84.55)	782,422 (84.54)	609,324 (93.02)
yes	990,152 (6.68)	784,470 (5.98)	16,924 (15.45)	143,067 (15.46)	45,691 (6.98)
Region					
HIV low-risk region	6,642,948 (44.84)	5,948,862 (45.32)	44,918 (41.00)	372,048 (40.20)	277,120 (42.31)
HIV medium-risk region	5,703,594 (38.50)	5,064,045 (38.58)	39,843 (36.37)	354,462 (38.30)	245,244 (37.44)
HIV high-risk region	2,469,758 (16.67)	2,113,327 (16.10)	24,801 (22.64)	198,979 (21.50)	132,651 (20.25)

F−M−: both were free of HBsAg; F+M+: both were HBsAg positive; F−M+, only husband was HBsAg positive; F+M−: only wife was HBsAg positive.

**Table 2:** Multivariate Logistic Regression for HBsAg Positivity in Couples.

	AOR for F+M+	95% CI	AOR for F−M+	95% CI	AOR for F+M−	95% CI
Wife’s age						
20–24 years	Ref		Ref		Ref	
25–29 years	1.01*	1.00,1.03	0.98***	0.98,0.99	1.02***	1.02,1.03
30–49 years	0.93***	0.92,0.95	0.95***	0.94,0.95	1.05***	1.04,1.05
Wife’s education						
≥college	Ref		Ref		Ref	
senior school	1.05***	1.02,1.07	0.93***	0.92,0.94	1	0.99,1.01
junior school	0.96***	0.94,0.98	0.83***	0.82,0.83	0.95***	0.94,0.96
≤primary school	1.03*	1.00,1.06	0.85***	0.84,0.86	0.99*	0.98,1.00
Couple’s syphilis	2.69***	2.57,2.82	1.73***	1.69,1.76	1.54***	1.50,1.58
HIV low-risk region	Ref		Ref		Ref	
HIV medium-risk region	1.03***	1.02,1.05	1.11***	1.10,1.11	1.03***	1.03,1.04
HIV high-risk region	1.53***	1.51,1.56	1.50***	1.49,1.51	1.34***	1.33,1.35

AOR: adjusted odds ratio; CI: confidence interval; couples were analytical units; each model was adjusted for same set of covariates. One star after the number means P < 0.05, and three stars means P < 0.001.
